# Clinical characteristics and related influencing factors of common rheumatic diseases concomitant with tuberculosis

**DOI:** 10.3389/fpubh.2022.948652

**Published:** 2023-01-16

**Authors:** Guo Tang, Xixi Chen, Yaxin Han, Qing Peng, Jiajun Liu, Yan Liu, Hongmei Guo, Xiaodan Wu, Jian Liu, Qiao Zhou, Li Long

**Affiliations:** ^1^Department of Nephrology, Bishan Hospital of Chongqing Medical University, Chongqing, China; ^2^Department of Rheumatology and Immunology, Sichuan Provincial People's Hospital, University of Electronic Science and Technology of China, Chengdu, China; ^3^Department of Rheumatology and Immunology, Chinese Academy of Sciences Sichuan Translational Medicine Research Hospital, Chengdu, China; ^4^Department of Rheumatology and Immunology, The People's Hospital of WenJiang, Chengdu, China; ^5^Department of Rheumatology and Immunology, Chengdu Second People's Hospital, Chengdu, China; ^6^Department of Nephrology and Rheumatology, The First Clinical Medical College, Zunyi Medical University, Zunyi, Guizhou, China; ^7^Department of Rheumatology and Immunology, Clinical Medical College, University of Electronic Science and Technology of China, Chengdu, China

**Keywords:** clinical characteristics, rheumatic diseases, tuberculosis, concomitant, tuberculin skin test, interferon gamma release assay

## Abstract

**Objective:**

To explore the clinical characteristics and risk factors of common systemic rheumatism concomitant with tuberculosis (TB).

**Methods:**

A total of 3,906 patients of RA, SLE, and SS diagnosed in the People's Hospital of Sichuan Province from January 2007 to January 2017 were collected. One hundred and five patients with TB were included as TB group, including 42 RA, 41 SLE, and 22 SS patients. In the non-TB group, 84 RA, 82 SLE, and 44 SS patients were randomly selected during the same period.

**Results:**

Fever was the most common symptom among RA, SLE, and SS patients with TB, accounting for 83.3%, 92.7%, and 68.2%, respectively. Cough, weight loss or fatigue were the next common. RA patients with TB were mostly pulmonary TB (PTB), accounting for 64.3%. The proportion of PTB for SLE and SS were 46.3%, 59.01%, respectively. In TB group, 59% RA, 57% SLE, and 62% SS with PTB had two or more chest CT findings. There were 48 TB cases received both Interferon Gamma Release Assay (IGRA) and Tuberculin skin test (TST) with positive rates of 91.8%, 45.8%, respectively. The daily average dose of glucocorticoids within 1 year in TB group was higher than that in non-TB group of SLE patients, lower counts of CD4+ T cell count were found in TB group (*P* < 0.05), while no such differences were found in RA and SS patients.

**Conclusion:**

RA patients with TB are mainly pulmonary TB. For SLE and SS patients, the chance of PTB and extrapulmonary tuberculosis is similar. Daily average dose of glucocorticoids within 1 year may be a common risk factor for RA, SLE and SS patients developing TB. Decreased CD4+ T cell count may also be a risk factor for SLE patients with TB. Symptoms of RA, SLE, SS with TB, are similar with the primary disease or other infection. It is recommended to conduct both TST and IGRA to help diagnose TB.

## 1. Introduction

Rheumatoid arthritis (RA), systemic lupus erythematosus (SLE), and Sjögren's syndrome (SS) are common rheumatic diseases with high morbidity. Glucocorticoids and immunosuppressants are required for treating these diseases, resulting in high infection risk and mortality. The increased comprehension of infections has led to rapid diagnosis and reasonable treatment of common pathogenic infections. Therefore, most infections can be effectively controlled at an early stage. However, the diagnosis and differentiation of infections caused by specific pathogens are difficult in clinical practice.

Tuberculosis (TB) is an infectious disease caused by the exposure of the body to Mycobacterium tuberculosis (Mtb). The lung is the most commonly affected organ; however, other organs can also be affected, leading to extrapulmonary tuberculosis. Since its advent, TB has been one of the major public health concerns worldwide. As of 2017, TB remained one of the top 10 leading causes of death worldwide. According to the World Health Organization, at least 10 million new cases of TB were reported worldwide in 2017; however, only 6.4 million new TB cases were officially reported by national authorities worldwide; the gap between the estimated and reported numbers is mainly owing to under-reporting and missed diagnoses (1). China, as a developing country with a large population base, experiences a high TB-related economic and social burden. Although the incidence of TB in China has decreased in recent years, it remains among the highest in the world; in addition, multidrug-resistant *Mycobacterium tuberculosis* infections, low patient attendance and poor medication adherence among patients with infection (2) are major concerns in China. Therefore, early diagnosis and prompt standardized treatment of TB are crucial for controlling the progression and spread of TB and improving patient prognosis (1, 3).

Patients with systemic rheumatic diseases (SRDs) are at high risk for TB (4–7). According to statistics, the incidence of TB in patients with RA is 0.23% (8). In addition, the incidence of TB in patients with SLE is 7 times higher than that in the healthy population in developed countries and is estimated to be 5% in developing countries (9). Similarly, the incidence of TB in patients with SS is higher than that in the healthy population (10). Because most SRDs exhibit atypical manifestations of TB, they are often considered pure rheumatic diseases, making TB diagnosis difficult and leading to a high misdiagnosis and missed diagnosis rate and delayed treatment. Therefore, improving clinical comprehension of SRDs concomitant with TB and the rational use of TB screening methods are essential to improve patient prognosis (11, 12).

Patients with RA, SLE and SS concomitant with TB diagnosed in Sichuan Provincial People's Hospital between January 2007 and January 2017 were included in this study to analyse the clinical manifestations and characteristics of TB. Subsequently, their clinical data were compared with those of patients with the corresponding rheumatic diseases without TB to identify risk factors for TB based on disease activity, treatment regimen and immune status of the body.

A few systematic studies have been reported on rheumatic diseases concomitant with TB; however, most were single-disease studies and included few observational indicators (13–19). In this study, we investigated the clinical manifestations and influencing factors of TB among patients with various common rheumatic diseases. Overall, the findings have important implications for a systematic understanding of the clinical characteristics and high-risk factors of rheumatic diseases concomitant with TB, which may assist in early diagnosis and treatment.

## 2. Materials and methods

Patients with RA, SLE and SS diagnosed in a hospital from January 2007 to January 2017 were retrospectively included based on the current internationally accepted classification criteria (20–22). The exclusion criteria were as follows: (1) the presence of two or more rheumatic diseases; (2) the presence of neoplastic diseases.

Patients with RA, SLE and SS who met the criteria for active TB (23, 24) were screened to examine the incidence of TB. Further, patients with other bacterial, fungal and viral infections were excluded from this group and served as the TB group.

Patients who met the inclusion criteria and were free of infection were randomly selected based on age in a 1:2 ratio among patients with rheumatic diseases hospitalized during the same period. Consequently, 84 patients with RA, 82 patients with SLE and 44 patients with SS were included in the RA, SLE and SS control groups, respectively (collectively referred to as the control group). The activity of SLE, RA and SS was assessed using the SLEDAI-2000, DAS28 and ESSDAI scales respectively.

General and clinical data were collected in detail. Characteristics of TB group were described, and differences between the TB group and the control group were compared.

The SPSS Statistics (version 22.0) software was used for analyses. The measurement data conforming to normal distribution including erythrocyte sedimentation rate (ESR) in SLE and SS patients, C-reactive protein (CRP) levels, rheumatoid factor (RF) titers, anti-cyclic citrullinated peptide antibody (anti-CCP) titers, CD4+T cell count, blood cell count, immunoglobulin G (IgG), Complement C3, Complement C4, average daily dose of glucocorticoids, treatment duration of glucocorticoid, and disease activity score were expressed as $\bar{x}$ ± s, and the *t*-test was used for comparing data between two groups. The measurement data not conforming to normal distribution including disease course and ESR in RA patients were expressed as M (P25 and P75), and a non-parametric test (Mann–Whitney U rank-sum test) was used for comparing data between two groups. Enumeration data including gender, positive rates of various antibodies, proportion of patients using immunosuppressants were expressed as frequencies or percentages, and the chi-square test or Fisher's exact probability method was used for comparing data between two groups. A paired Chi-square test was used to compare the positive rates of interferon-gamma release assay (IGRA) and tuberculin skin test (TST) in patients with TB infection. A *P* < 0.05 indicated a significant difference.

## 3. Results

### 3.1. General information

A total of 3906 patients with RA, SLE and SS diagnosed from January 2007 to January 2017 met the inclusion criteria, including 1,273 patients with RA, 1,393 patients with SLE and 1,240 patients with SS. Of the 3,906 patients, 297 (79 patients with RA, 94 patients with SLE and 37 patients with SS) developed TB, with infection rates of 6.21%, 6.75%, and 2.98%, respectively. In addition, the TB group was excluded from other pathogenic infections. The general information of patients in the TB group is shown in Table 1.

**Table 1:** General information of patients in the TB group.

		**TB group**	
	**RA with TB**	**SLE with TB**	**SS with TB**
Sex (F/M)	21/21	35/6	17/5
Age (y), mean ± SD	54.6 ± 14.1	40.2 ± 14.4	53.3 ± 14.6
Disease course (m), median, range	48 (12, 120)	36 (12, 66)	12 (1, 30)
DAS28, mean ± SD	5.65 ± 1.40	–	–
SLEDAI, mean ± SD	–	7.08 ± 6.13	–
ESSDAI, mean ± SD	–	–	2.79 ± 1.65

### 3.2. Clinical manifestations of various common rheumatic diseases concomitant with TB

The main clinical manifestations of patients with RA in the TB group were fever (83.3%), cough (69%) and weight loss (45.2%); those of patients with SLE were fever (92.7%), cough (63.4%) and weight loss (48.8%) and those of patients with SS were fever (68.2%), cough (54.5%) and malaise (40.9%). Fever was the predominant manifestation in patients with either of the three diseases, followed by cough. All patients with previously diagnosed TB were treated regularly and cured (Table 2).

**Table 2:** Clinical manifestations in the TB group.

**Clinical manifestations**	**RA (*n* = 42)**	**SLE (*n* = 41)**	**SS (*n* = 22)**
**Fever, *n* (%)**	**35 (83.3)**	**38 (92.7)**	**15 (68.2)**
Cough, *n* (%)	29 (69.0)	26 (63.4)	12 (54.5)
Haemoptysis, *n* (%)	10 (23.8)	8 (19.5)	2 (9.1)
Malaise, *n* (%)	15 (35.7)	17 (41.4)	9 (40.9)
Night sweat, *n* (%)	15 (35.7)	12 (29.3)	7 (31.8)
Weight loss, *n* (%)	19 (45.2)	20 (48.8)	6 (27.3)
Lymphonodular hyperplasia, *n* (%)	7 (16.6)	8 (19.5)	4 (18.2)
Previously diagnosed TB, *n* (%)	5 (11.9)	4 (9.8)	5 (22.7)
Cured TB, *n* (%)	5 (11.9)	4 (9.8)	5 (22.7)

TB, Tuberculosis; RA, Rheumatoid arthritis; SS, Sjögren's syndrome; SLE, systemic lupus erythematosus.

### 3.3. Involvement sites of common rheumatic diseases concomitant with TB

Of the 42 patients with RA in the TB group, pulmonary and extrapulmonary tuberculosis accounted for 64.3% and 14.3% cases, respectively. Of the 41 patients with SLE in the TB group, pulmonary and extrapulmonary tuberculosis accounted for 46.3% and 39.0% cases, respectively. Tuberculous meningitis was the predominant form of extrapulmonary tuberculosis and was observed in 7 (17.1%) patients, including 1 patient with concomitant pulmonary tuberculosis. Of the 22 patients with SS in the TB group, pulmonary and extrapulmonary tuberculosis accounted for 59.1% and 40.9% cases, respectively, and tuberculous pleurisy was the predominant form of extrapulmonary tuberculosis (13.6%) (Table 3).

**Table 3:** Distribution of TB foci in the TB group.

**TB foci**	**RA with TB**	**SLE with TB**	**SS with TB**
Pulmonary TB, *n* (%)	27 (64.3)	19 (46.3)	13 (59.1)
Tuberculous meningitis, *n* (%)	0	6 (14.6)	0
Tuberculous pericarditis, *n* (%)	0	0	1 (4.5)
Tuberculous pleuritis, *n* (%)	4 (9.5)	5 (12.2)	3 (13.6)
Tuberculous peritonitis, *n* (%)	1 (2.4)	1 (2.4)	0
Joint tuberculosis, *n* (%)	6 (14.3)	4 (9.8)	1 (4.5)
Tuberculous lymphadenitis, *n* (%)	2 (4.8)	0	0
Two or more foci, *n* (%)	0	3 (7.3)	2 (9.1)
Unknown involvement sites, *n* (%)	2 (4.8)	3 (7.3)	2 (9.1)

TB, Tuberculosis; RA, Rheumatoid arthritis; SS, Sjögren's syndrome; SLE, Systemic lupus erythematosus.

All patients with pulmonary TB underwent computed tomography (CT) of the chest. The proportion of patients with RA, SLE and SS who had a typical involvement site of TB (post-apical segment of the upper lobe or dorsal segment of the lower lobe) was 33.3%, 42.9%, and 53.3%, respectively. A total of 11 (40.7%) patients with RA had only one imaging manifestation of TB, mostly in the form of plaque, fibrous streak or nodules. A total of 9 (42.9%) patients with SLE had only one imaging manifestation of TB; of which, 7 patients had plaques, fibrous streaks or nodules; 1 patient had tuberculoma and 1 patient had miliary TB. A total of 5 (33.3%) patients with SS had only one imaging manifestation of TB; of which, 3 patients had plaques, fibrous streaks or nodules and 2 patients experienced interstitial changes. In patients with ≥2 imaging manifestations, calcified foci, masses or cavities were common (Table 4).

**Table 4:** Chest CT manifestations of pulmonary TB in TB group.

**Diseases**	**Involvement sites**, ***n*** **(%)**		**Single lesion, *n* (%)**	**Two or more lesions, *n* (%)**
	^*^ **Tipical sites**	**Other sites**		
RA with TB	9 (33.3)	18 (66.7)	11 (40.7)	16 (59.3)
SLE with TB	9 (42.9)	12 (57.1)	9 (42.9)	12 (57.1)
SS with TB	8 (53.3)	7 (46.7)	5 (33.3)	10 (66.7)

* Post-apical segment of the upper lobe or dorsal segment of the lower lobe.

TB, Tuberculosis; RA, Rheumatoid arthritis; SS, Sjögren's syndrome; SLE, Systemic lupus erythematosus; CT, Computed tomography.

### 3.4. Comparison of IGRA and TST results

Of the 105 patients with TB infection, 48 underwent both IGRA and TST. Of these patients, 20 (41.7%) had positive results for both tests (10 patients had strongly positive TST results), 24 (50.0%) had positive results for IGRA only and 2 (4.2%) had positive results for TST only (1 patient had a strongly positive result). The rate of IGRA positivity (91.8%) was greater than that of TST positivity (45.8%) (*P* < 0.05).

### 3.5. Diagnostic methods

Positive smear or culture results for Mtb were the typical outcomes observed in patients with pulmonary TB (62.7%). TB in other sites was mostly diagnosed based on clinical indications (e.g., imaging changes and/or effective diagnostic anti-TB treatment). Joint tuberculosis was mostly observed among patients with RA with extrapulmonary TB (14.3%), with imaging findings suggestive of focal worm-bitten and broken glass-like and other manifestations in 5 of the 6 patients with joint tuberculosis. A total of 3 patients were clinically diagnosed by imaging; of which, 1 patient had a positive result for acid-fast bacillus (AFB) smear examination of arthrocentesis fluid and 2 patients had positive pathological biopsy results. Among patients with SLE with extrapulmonary TB, the proportion of patients with tuberculous meningitis was highest (14.6%). Cerebrospinal fluid was positive for AFB smear in 1 (14.3%) patient, positive for culture in 1 (14.3%) patient and positive for both smear and culture in 1 (14.3%) patient. The abnormal manifestations of tuberculous meningitis mainly included elevated total protein levels (71.3%) and decreased glucose levels (71.3%). Additionally, tuberculous pleuritis was mostly observed among patients with SS with extrapulmonary TB (13.6%), with 1 patient diagnosed based on pleural biopsy and 2 patients diagnosed based on clinical indications.

Summing up, samples from 98 (93.3%) patients were stained with AFB and cultured *in vivo*. Among which, 14 cases (14.3%) were positive for AFB smear only, 7 (7.1%) were positive for Mtb culture only, and 12 (12.2%) had positive results in both AFB smear and Mtb culture. No drug-resistant tuberculosis was found.

### 3.6. Treatment and prognosis

All patients were administered triple (isoniazid, rifampicin, and pyrazinamide) or quadruple (isoniazid, rifampicin, pyrazinamide, and ethambutol) antituberculosis treatment immediately after the diagnosis of TB. The condition of all 108 patients in the TB group improved after treatment, with no deaths.

## 4. Factors influencing the incidence of tuberculosis in patients with common rheumatic diseases

### 4.1. Comparison of clinical data between the TB and control groups

Differences in sex, age, disease course and disease activity between the TB and control groups were not statistically significant (*P* > 0.05). ESR were 71.5 mm/h (52.0, 104.8) and 53.0 mm/h (28.5, 80.0) in the TB (*n* = 42) and RA control (*n* = 84) groups, respectively (*P* < 0.05), whereas CRP levels were 23.0 mg/L (7.5, 80.5) and 20.3 mg/L (7.6, 42.0) in the TB (*n* = 42) and RA control (*n* = 84) groups, respectively (*P* < 0.05). No statistically significant differences were observed in other indicators such as blood cell count, RF, CCP titer, Ig and CD4+ T cell count.

In the TB (*n* = 41) and SLE control (*n* = 82) groups, ESRs were 53.7 ± 29.0 mm/h (52.0, 104.8) and 40.7 ± 28.3 mm/h (28.5, 80.0), respectively (*P* < 0.05), whereas CRP levels were 49.0 mg/L (13.5, 90) and 12.8 mg/L (6.0, 34.8), respectively (*P* < 0.05). No statistically significant differences were observed in other indicators such as blood cell count, ANA, ENA profile, Ig and complement.

In the TB (*n* = 22) and SS control (*n* = 44) groups, ESRs were 79.6 ± 34.8 mm/h and 44.7 ± 31.2 mm/h, respectively (*P* < 0.05), whereas CRP levels were 18.5 mg/L (9.5, 27.8) and 1.0 mg/L (0.0, 6.0), respectively (*P* < 0.05). No statistically significant differences were observed in other indicators such as blood cell count, RF, ANA, ENA profile (e.g., SSA and SSB antibodies), complement, Ig and CD4+ T cell count.

### 4.2. Medication administration in patients in the TB and non-TB (control) groups

The administration of glucocorticoids and immunosuppressants was compared between the TB and non-TB groups. The average daily dose of glucocorticoids was higher in all three rheumatic disease groups (TB group) than in the non-TB group (*P* < 0.05) (Table 5). The immunosuppressants used in patients with RA included methotrexate, leflunomide, cyclophosphamide and hydroxychloroquine; those used in patients with SLE included cyclophosphamide, cyclosporine A, mycophenolate mofetil, tacrolimus and leflunomide and those used in patients with SS included aminopterin, leflunomide, cyclophosphamide and hydroxychloroquine.

**Table 5:** Medication administration in the TB and non-TB groups.

		**TB group**	**Non-TB group**	***P***
^*^ADD of glucocorticoid only (mg/d), mean ± SD	RA	15.8 ± 9.2	6.9 ± 4.4	0.03
	SLE	0	0	–
	SS	15.0 ± 7.1	5.8 ± 1.5	0.04
^*^ADD of glucocorticoid combined with immunosuppressants (mg/d), mean ± SD	RA	13.9 ± 6.3	7.42 ± 4.7	0.01
	SLE	17.8 ± 6.8	10.7 ± 7.9	0.03
	SS	13.0 ± 5.7	5.2 ± 4.4	0.04
Methyprednisone pulse therapy performed within 1 year, *n* (%)	RA	0	0	–
	SLE	6, 14.6	8, 9.7	0.39
	SS	0	0	–
Treatment duration of glucocorticoid (months), mean ± SD/median, range	RA	60 (1, 120)	60 (1, 120)	0.95
	SLE	27.2 ± 16.9	30.56 ± 28.5	0.53
	SS	34 (6.5, 64)	32 (4, 78)	0.86
Use of immunosuppressants, *n* (%)	RA	20, 47.6	54, 64.3	0.07
	SLE	34, 82.9	74, 90.2	0.24
	SS	7, 31.8	10, 22.7	0.43

* Converted into dose of prednisone.

TB, Tuberculosis; RA, Rheumatoid arthritis; SS, Sjögren's syndrome; SLE, Systemic lupus erythematosus; ADD, Average daily dose.

## 5. Discussion

Patients with common rheumatic diseases are at an increased risk of developing opportunistic infections owing to abnormal regulation of autoimmune function, disease activity-caused body weakness and the use of immunosuppressants. A study reported considerable morbidity and mortality of infections in patients with common rheumatic diseases (25). Among these patients, the risk of TB is 4 and 5 times higher in patients with RA and SLE, respectively, than in the healthy population (26, 27). In this study, we retrospectively analyzed the clinical characteristics and laboratory test indicators of patients with RA, SLE and SS concomitant with TB who were diagnosed in Sichuan provincial People's Hospital in the past decade. The results of this study may assist in early diagnosis and treatment.

Common rheumatic diseases may manifest as fever, malaise and weight loss, which are highly similar to the manifestations of TB activity. Therefore, common rheumatic diseases may be misdiagnosed as a general infection, leading to a very high misdiagnosis and underdiagnosis rate. In this study, the most common manifestations of RA (or SLE) concomitant with TB were fever (83.3%), cough (69%) and weight loss (45.2%); the main manifestations of SLE concomitant with TB were fever (92.7%), cough (63.4%), and weight loss (48.8%). In addition, patients with SS concomitant with TB mainly presented with fever (68.2%), cough (54.5%) and malaise (40.9%). Fever was the most common manifestation of the three conditions. Furthermore, the proportion of patients with night sweats and haemoptysis was 35.7% and 23.8%, 29.3% and 19.5%, and 31.8% and 9.1%, respectively, among patients with RA, SLE and SS, respectively, which was consistent with the results of previous studies (28).

In terms of foci distribution, pulmonary TB was predominant in patients with RA and SS (64.3%). In addition, the proportion of patients with pulmonary and extrapulmonary TB was comparable among patients with SLE and SS. The proportion of patients with RA, SLE and SS who had a typical involvement site of TB (post-apical segment of the upper lobe or dorsal segment of the lower lobe) was 33.3%, 42.9%, and 46.2%, respectively. Chest imaging suggested diverse manifestations. The proportions of patients with RA, SLE and SS with ≥2 imaging manifestations were 59%, 57%, and 62%, respectively. Moreover, fibrous plaques, fibrous streaks and nodules accounted for a larger proportion of presentations, which increased the difficulty in differentiating TB from other pathogenic infections and primary disease lung involvement. Therefore, multiple imaging examinations, early bronchoscopy, CT-guided puncture and endoscopic surgical biopsy may be helpful for early diagnosis.

In this study, patients with RA with extrapulmonary TB most often presented with joint tuberculosis. A total of 6 patients with joint tuberculosis mostly presented with swelling or pain in multiple joints, including tuberculosis-involved joints, as the main clinical manifestation instead of swelling and pain in tuberculosis-involved joints only, which may be confused with pain in multiple joints caused by a primary disease. Therefore, active imaging can provide diagnostic clues. Furthermore, patients with SLE with extrapulmonary TB most often presented with tuberculous meningitis. Abnormal cerebrospinal fluid manifestations mainly included elevated total protein (71.3%) and decreased glucose (71.3%) levels. However, the rate of positive smear and culture for tuberculous meningitis was very low, making it difficult to differentiate it from neuropsychiatric lupus. A total of 7 patients in this study presented with fever and headache, and cerebrospinal fluid examination suggested increased protein and decreased glucose levels. Of the 7 patients, two had been misdiagnosed as having active lupus and were subjected to anti-TB treatment after the failure of glucocorticoid therapy, eventually achieving effective clinical outcomes. Therefore, if a patient with SLE presents with fever and headache after receiving increased doses of glucocorticoids or immunosuppressive therapy and the cerebrospinal fluid examination suggests elevated total protein and decreased glucose levels, tuberculous meningitis may be present. In this study, extrapulmonary TB was predominant in patients with SS with tuberculous pleurisy (13.6%), which is clinically diagnosed mainly *via* the absorption of pleural fluid and the relief of symptoms such as fever and cough after anti-TB treatment.

No significant differences in sex, age and disease course were observed between the TB and non-TB groups, which is consistent with the results of previous studies (29). Risk factors for the incidence of TB in patients with SLE have been explored more frequently. The average daily dose of glucocorticoids is associated with the incidence of TB, with conflicting findings regarding the correlation between the administration of immunosuppressants and disease activity (30–32). In this study, the average daily dose of glucocorticoids was significantly higher in the TB group than in the control group, suggesting that it is a high-risk factor for the incidence of TB, which is consistent with the findings of previous studies (33). Although disease activity and the administration of immunosuppressants were not found to be associated with the risk of TB, a decrease in CD4+ T cell count increased the risk of TB. To the best of our knowledge, the correlation between CD4+ T cell count and the risk of TB in patients with SLE has not been reported in previous studies. This study suggests that CD4+ T cell count can help to predict the risk of TB. Therefore, large-scale prospective trials should be designed in the future to validate this finding.

To date, studies on TB in patients with RA have focused on biological agents, especially TNF-α antagonists. Biological agents, especially monoclonal TNF-α antagonists, increase the risk of TB compared with conventional disease-modifying antirheumatic drugs (DMARDs). However, Arkema et al. (34) reported that although the incidence of TB in patients with RA receiving TNF-α antagonists was consistently higher than that in patients who were not treated with biological agents, it significantly decreased each year after 2002. However, the incidence of TB in patients with RA who were not treated with biological agents remained approximately 4 times higher than that in the general population, with no trend of improvement. Therefore, the risk of TB caused by high-risk biological agents can be reduced through rigorous TB screening and aggressive prophylactic anti-TB treatment. However, the population not receiving biologics is neglected to some extent, resulting in a poorly controlled risk of TB. In this study, patients in both TB and control groups were not administered biological agents. Similar to the case of SLE, the average daily dose of glucocorticoids was associated with the incidence of TB in patients with RA, whereas the use of conventional DMARDs was not significantly associated with the risk of TB. Moreover, indicators such as disease activity and CD4+ T cell count were not associated with the risk of developing TB in patients with RA.

A few studies have been conducted to quantify the risk of TB in patients with SS. Chang et al. (16) observed a positive correlation between the average daily use of glucocorticoids and the incidence of TB based on a survey of a Taiwanese population. However, he did not observe a correlation between immunosuppressants and the incidence of TB and did not investigate the association between disease activity and the risk of TB. In this study, the average daily dose of glucocorticoids was correlated with the incidence of TB in patients with SS, whereas indicators such as disease course, disease activity, IgG and CD4+ T cell count were not correlated with the incidence of TB. However, owing to the relatively small number of patients with SS with TB, future expansion of the sample size is warranted to validate this finding.

In clinical practice, differential diagnosis of Mtb and non-tuberculous mycobacteria (NTM) species is still a challenge since both Mtb and NTM show positivity to AFB smear and have similar clinical manifestations. However, NTM diseases have poor response to anti-TB drugs (35). In our study, although no NTM culture or gene sequencing was made, anti-tubercular treatment was effective for every patient in TB group, which in turn confirmed the diagnosis.

Overall, in terms of the risk of antirheumatic therapy-induced TB, the average daily dose of glucocorticoids administered to patients with RA, SLE and SS concomitant with TB was more than a prednisolone equivalent dose of 10 mg/d, and the maximum dose received by patients with SLE was 17.8 ± 6.8 mg/d. A study by Kim HA (36) suggested that the risk of TB was significantly increased when patients with rheumatic diseases received an average daily prednisone dose of 10–20 mg, which is consistent with the results of the present study. Therefore, patients with rheumatic diseases who require long-term use of equivalent prednisone at a dose of >10 mg/d should be alerted to the incidence of TB. In addition, pre-administration screening, continuous follow-up and prophylactic anti-TB treatment, if required, are necessary to reduce the risk of TB incidence or recurrence. Given the complexity of TB manifestations, TB should be screened at all possible involvement sites if anti-rheumatic therapy did not achieve the desired efficacy. Furthermore, combined testing *via* IGRA and TST is recommended to provide additional diagnostic clues.

## Data availability statement

The original contributions presented in the study are included in the article/supplementary material, further inquiries can be directed to the corresponding authors.

## Author contributions

Conceptualization: GT and XC. Methodology: QP. Software: YH. Validation: JiajL, QP, and HG. Formal analysis: JianL. Investigation and resources: YL. Data curation: XW. Writing—original draft preparation: QZ. Writing—review and editing: QZ and LL. Visualization: GT. Supervision: XC. Project administration: LL. All authors have read and agreed to the published version of the manuscript.
